# Supplementary material for: Disturbance modifies payoffs in the explore-exploit trade-off
Source: Nat Commun. 2019 Jul 29;10:3363. doi: 10.1038/s41467-019-11106-y (PMC6662700; doi:10.1038/s41467-019-11106-y)
Supplement: Supplementary file 1 — Supplementary Information [file 41467_2019_11106_MOESM1_ESM.pdf]

Supplementary Information

Supplementary Figures

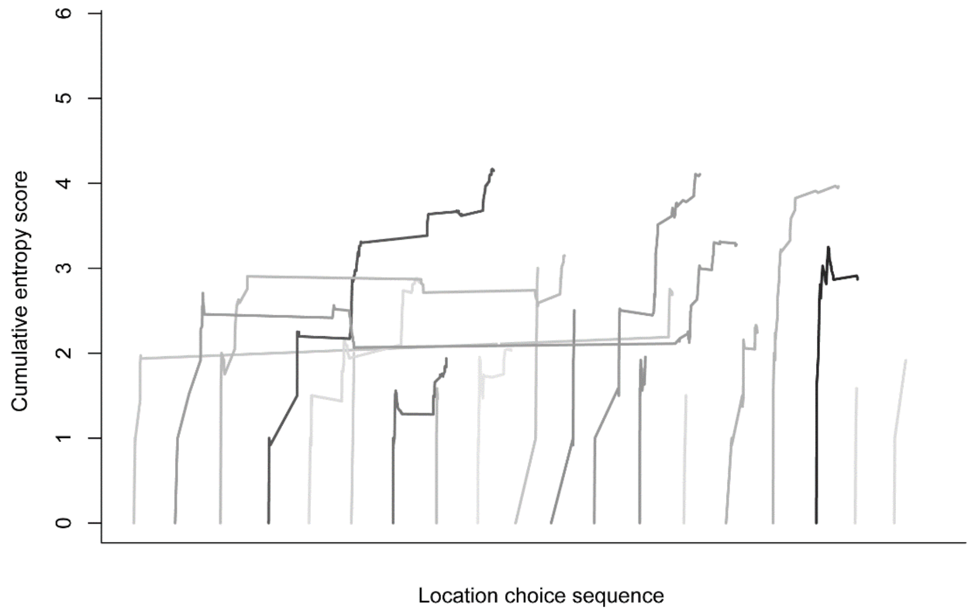

Supplementary Figure 1. Deleted vessel records. Entropy trajectories for 19 vessels with statistically outlying trajectory slopes. Trajectories have been staggered horizontally to improve clarity. The horizontal axis shows the choice sequence and the vertical axis shows the entropy score. The erratic trajectories are caused by sparse data and the vessels were removed from the analysis.

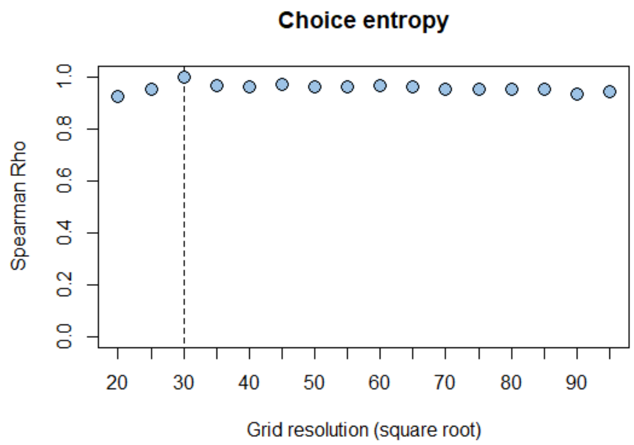

Supplementary Figure 2. To test the sensitivity of our results to the chosen raster size (30 x 30 = 900 cells), we recalculate choice entropy using grids ranging from 20 x 20 = 400 to 95 x 95 = 9025 cells, which represents more than an order of magnitude difference in scale. Blue markers show Spearman correlation scores (rho) between our chosen resolution (dashed vertical line) and the other resolutions. Correlation is extremely high (mean rho = 0.96; sd = 0.016; for all tests, $P < 0.001$) between our chosen resolution and all others, indicating that our calculations are not particularly sensitive to raster size.

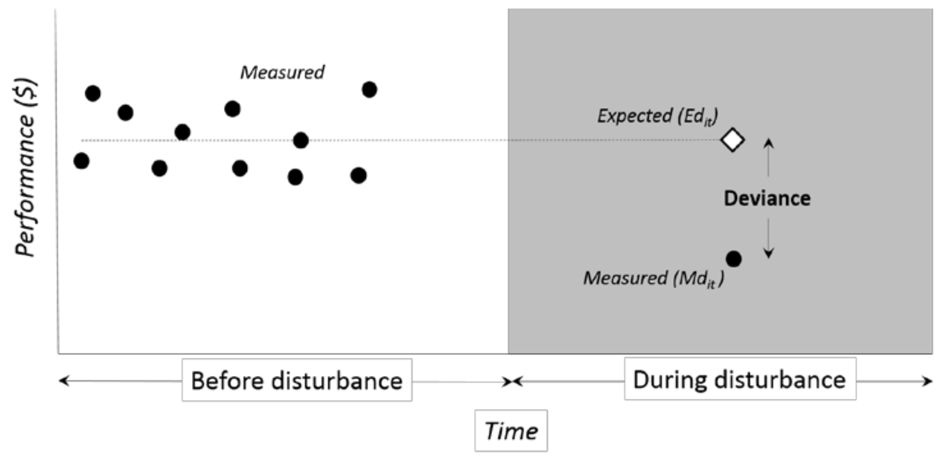

Supplementary Figure 3. Conceptual diagram of modeling framework. Most vessels will experience negative deviances in performance during a major disturbance, so our hypothesis is formulated such that more-exploratory vessels are predicted to experience less-negative deviances. In the illustration, solid circles represent measured performance and the open diamond represents expected performance under a business-as-usual scenario. The dashed line represents P_i, or the mean pre-disturbance performance of vessel i while controlling for the confounding variables trip duration and contemporaneous fleet performance. The expected performance of vessel i on trip t during the disturbance, Ed_{it}, is then projected and compared against the measured performance, Md_{it} to quantify the deviance in performance ΔP_{it}. Specifically, ΔP_{it} is standardized for each vessel by dividing Md_{it} by Ed_{it}, then 1 is subtracted to scale the deviance relative to zero so that positive/negative values indicate better/worse than business-as-usual performance. In the illustration, if Md_{it} = \$500 and Ed_{it} = \$1000, then ΔP_{it} = (500/1000) – 1 = -0.5, meaning that vessel i on trip t experienced a 50% decline relative to business-as-usual performance.

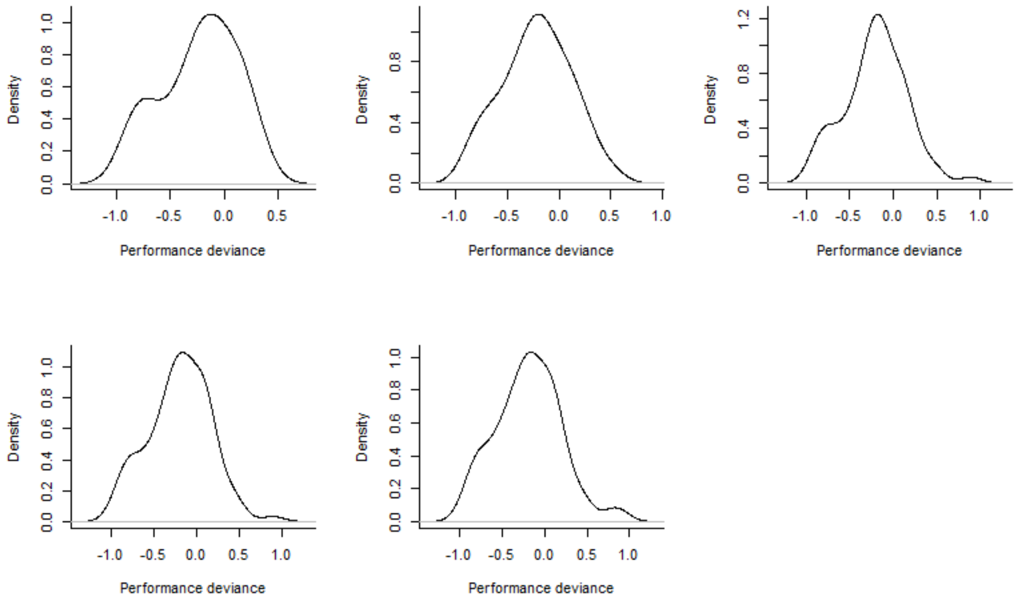

Supplementary Figure 4. Density plots of the post-disturbance response variable, performance deviance ΔP_i which quantifies each vessels' deviance from a projected 'business-as-usual' scenario. The variable is centered on zero such that worse than 'business-as-usual' performance has a negative value. The variable was used in N = 5 growing-window model fits (Figure 3D, main text), and the plots show that the values approximate continuous Gaussian distributions and are not piled against the bounds.

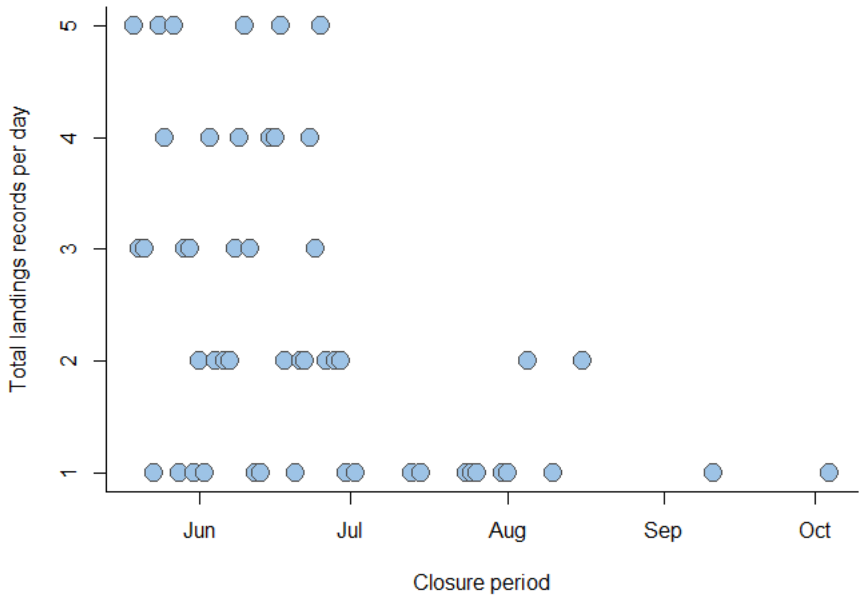

Supplementary Figure 5. Diminishing data availability. The number of longline fishing trips decayed rapidly during the closure, indicating that many vessels left the longline fishery, either by cessation of fishing or by switching to another gear type. The horizontal axis shows time after the start of the closure period and the vertical axis shows the total number of trips terminating on each day of the closure.

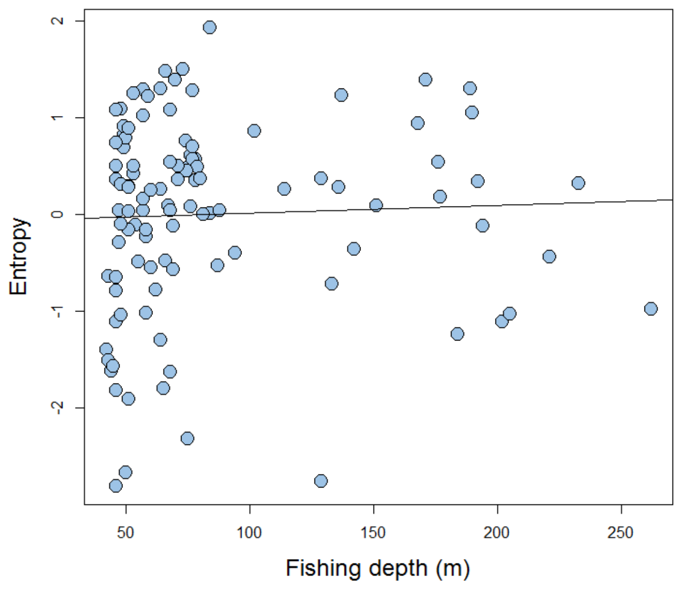

Supplementary Figure 6. Entropy score is not confounded by fishing depth. Entropy score (*S*) plotted against median fishing depth for each of the 106 vessels (round blue markers), showing that a range of EETO strategies is employed by both shallower- and deeper-water vessels. The line shows a linear model fitted to the data ($F_{[104]}$ = 0.1636; P = 0.687).

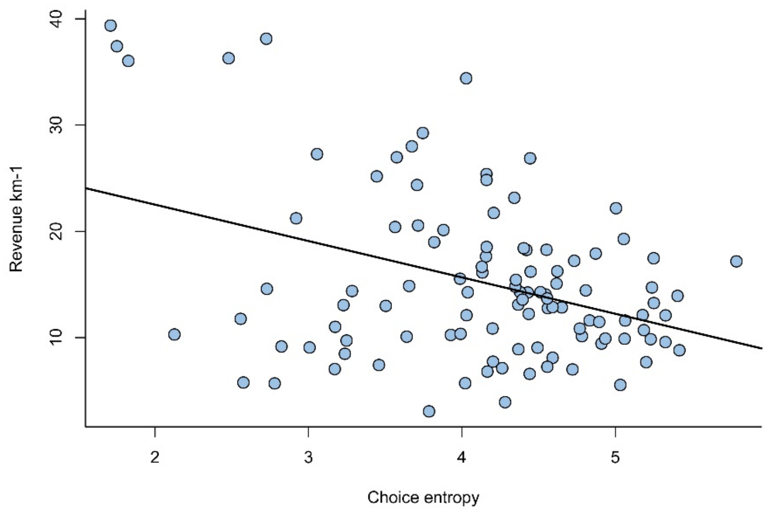

Supplementary Figure 7. Investment of resources in exploration introduces risks in terms of expected payoffs and likely requires additional travel costs relative to an exploitation strategy |To investigate whether vessels with higher choice-entropy EETO strategies incurred higher costs relative to revenues, we regress entropy score, *S*, on revenue per kilometer travelled. Blue markers show individual vessel scores. We find that higher entropy vessels travelled farther per unit of revenue ($F_{[104]}$ = 17.29, P < 0.001), confirming that there are measurable costs to investment in exploration.

Supplementary Table 1. Results from model fitting. Equations are fitted by stepwise selection using AICs and BICs, using centered covariates (i.e., expressed in standard deviations around zero means). The change in AIC/BIC is reported for dropped covariates, which are listed in order of deletion. Coefficient estimates are provided for covariates that are retained in optimal models. See Table 1 for details of covariates. Equation numbers as per main text.

Eqn. 5. $P_i = \alpha + \beta_1 S_i + \beta_2 S_i^2 + \beta_3 \text{prt}_i + \beta_4 \text{prt}_i^2 + \beta_5 \text{activity}_i + \beta_6 \text{length}_i + \varepsilon_i$

	Effect size	Standard error	*t*-value	*P*-value	δAIC	δBIC
prt	-	-	-	-	-1.99	-4.66
entropy, *S*	-	-	-	-	-1.99	-4.66
S^2	-	-	-	-	-0.82	-3.49
prt^2	-	-	-	-	-0.96	-3.62
intercept	10905	366.9	29.722	< 0.001	-	-
activity	1281.6	368.5	3.47	< 0.001	-	-
length	2302.1	368.7	6.244	< 0.001	-	-
	Degrees of freedom = 103					

Eqn. 6. Mixed effects model fitted to first 50 days of disturbance

$\Delta P_i = (\beta_0 + b_{0i}) + \beta_1 S_i + \beta_2 S_i^2 + \beta_3\text{prt}_i + \beta_4\text{prt}_i^2 + \beta_5\text{activity}_i + \beta_6\text{length}_i + \beta_7\text{displacement}_i + \varepsilon_i$

Random intercept, $b_{0i} \sim \text{Normal}(0, \sigma_i^2)$ (vessel ID as random factor)

	Effect size	Standard error	*t*-value	*P*-value	δAIC	δBIC
prt^2	-	-	-	-	-1.91	-4.5
S^2	-	-	-	-	-1.74	-4.32
activity	-	-	-	-	-1.8	-4.38
intercept	-0.343	0.053	-6.543	< 0.001	-	
entropy, *S*	0.107	0.067	1.603	0.115*	-	
length	0.056	0.041	1.387	0.171*	-	
displacement	-0.128	0.046	-2.767	0.008	-	
prt	-0.103	0.049	-2.062	0.044		
	Degrees of freedom = 50					

*AIC and BIC both support retaining in the model.

Eqn. 6b. As eqn. 6, but fitted to data subset from first 10 days of closure.

	Effect size	Standard error	*t*-value	*P*-value	δAIC	δBIC
S^2	-	-	-	-	-1.84	-3.06
prt^2	-	-	-	-	-0.33	-1.55
length	-	-	-	-	-0.38	-1.59

intercept	-0.576	0.087	-6.591	< 0.001	-	-
entropy, *S*	0.318	0.112	2.841	0.01	-	-
displacement	-0.178	0.066	-2.708	0.014	-	-
activity	0.134	0.062	2.168	0.042	-	-
prt	-0.125	0.092	-1.363	0.188*	-	-
	Degrees of freedom = 20					

*AIC and BIC both support retaining in the model.

Supplementary Table 2. Coefficient estimates for full models prior to simplification by stepwise selection.

Eqn. 5. $P_i = \alpha + \beta_1 S_i + \beta_2 S_i^2 + \beta_3 prt_i + \beta_4 prt_i^2 + \beta_5 \text{activity}_i + \beta_6 \text{length}_i + \varepsilon_i$

	Effect size	Std. error	*t*-value	*P*-value
intercept	10957.32	502.89	21.789	< 2e-16
S	19.03	475.56	0.040	0.96816
S^2	-277.38	293.71	-0.944	0.34727
length	2365.31	383.49	6.168	1.51e-08
activity	1270.15	411.73	3.085	0.00264
prt	-5.69	533.87	-0.011	0.99152
prt^2	225.42	240.34	0.938	0.35058

Residual standard error: 3813 on 99 degrees of freedom

Multiple R-squared: 0.3449, Adjusted R-squared: 0.3052

F-statistic: 8.688 on 6 and 99 DF, P-value: 1.344e-07

Eqn. 6. Mixed effects model fitted to first 50 days of disturbance

$\Delta P_i = (\beta_0 + b_{0i}) + \beta_1 S_i + \beta_2 S_i^2 + \beta_3 \text{prt}_i + \beta_4 \text{prt}_i^2 + \beta_5 \text{activity}_i + \beta_6 \text{length}_i + \beta_7 \text{displacement}_i + \varepsilon_i$

Random intercept, b_{0i} ~ Normal(0, σ_i^2) (vessel ID as random factor)

	Effect size	Std. error	*t*-value	*P*-value
intercept	-0.3563958	0.06553619	-5.438152	<0.001
S	0.1410449	0.09338898	1.510295	0.1377
S^2	-0.0332069	0.07170048	-0.463133	0.6454
activity	0.0268584	0.04631012	0.579969	0.5647
length	0.0496939	0.04251160	1.168950	0.2483
displacement	-0.1460333	0.05416513	-2.696076	0.0097
prt	-0.1034350	0.05733754	-1.803967	0.0776
prt^2	0.0099447	0.03623728	0.274434	0.7850

Number of Groups (vessels): 55 ___

Eqn 6b. As eqn 6 but fitted to data subset from first 10 days of closure.

	Value	Std. error	*t*-value	*P*-value
intercept	-0.4960826	0.11079406	-4.477520	<0.001
S	0.2021894	0.25325454	0.798365	0.4357
S^2	0.0635844	0.19497111	0.326122	0.7483
activity	0.1467663	0.06433732	2.281200	0.0357
length	-0.0712719	0.05769401	-1.235344	0.2335
displacement	-0.1996809	0.07480606	-2.669315	0.0162
prt	-0.1738744	0.10460381	-1.662218	0.1148
prt^2	-0.1066289	0.09877051	-1.079562	0.2954

Number of Groups (vessels): 25
